# Therapeutic Applications of Targeted Alternative Splicing to Cancer Treatment

**DOI:** 10.3390/ijms19010075

**Published:** 2017-12-28

**Authors:** Jung-Chun Lin

**Affiliations:** 1 School of Medical Laboratory Science and Biotechnology, College of Medical Science and Technology, Taipei Medical University, Taipei 110, Taiwan; lin2511@tmu.edu.tw; Tel.: +886-2-2736-1661 (ext. 3330); Fax: +886-2-2732-4510; 2 PhD Program in Medicine Biotechnology, College of Medical Science and Technology, Taipei Medical University, Taipei 110, Taiwan

**Keywords:** alternative splicing, oligonucleotide, small molecule

## Abstract

A growing body of studies has documented the pathological influence of impaired alternative splicing (AS) events on numerous diseases, including cancer. In addition, the generation of alternatively spliced isoforms is frequently noted to result in drug resistance in many cancer therapies. To gain comprehensive insights into the impacts of AS events on cancer biology and therapeutic developments, this paper highlights recent findings regarding the therapeutic routes of targeting alternative-spliced isoforms and splicing regulators to treatment strategies for distinct cancers.

## 1. Introduction

Alternative splicing (AS) constitutes a pivotal mechanism for expanding the transcriptome and proteome diversity, which provides evolutionary advantages to higher eukaryotes [1,2]. Approximately 95% of human genes transcribe more than one transcript through AS mechanisms to expand the genomic diversity [3,4]. AS is a spatiotemporal process that is critical for cell differentiation, organogenesis, and cell functions [5]. The interplay between *cis*-elements within the regulated gene and the *trans*-splicing regulator constitutes the major mechanism for the precise execution of AS events [6,7]. A growing body of studies has revealed different mechanisms, including mutations of regulatory elements of carcinogenesis-related genes [8,9] and altered expressions of core or accessory splicing factors [10,11], through which AS is perturbed, subsequently leading to carcinogenesis. These results suggest the therapeutic value for cancer treatment of targeting mutant *cis*-element or impaired *trans*-factor production. In this review, the manipulated mechanisms and pathological impacts of altered AS events on the initiation, maintenance, and drug resistance of distinct cancers is first highlighted. The potential applications of cancer-related AS events as targets for cancer therapy are next discussed.

### 1.1. The Regulatory Mechanism Involved in Alternative Splicing

Constitutive splicing is carried out to remove introns by the spliceosome machinery that is composed of five small nuclear ribonucleoproteins (snRNPs; U1, U2, U4, U5, and U6) and up to 300 other proteins [12]. However, the presence of canonical splice sites containing conserved sequences cannot efficiently trigger a specific splicing process [13]. *Cis*-elements provide additional information to manipulate the strength of splice sites, which consequently determines the utilization of regulated exons [14]. *Cis*-elements within pre-messenger (m)RNA recruit two major groups of *trans*-acting factors: serine/arginine-rich splicing factors (SRSFs) and heterogeneous nuclear (hn)RNPs. In general, SRSFs enhance the splicing process by interacting with exonic *cis*-elements, also referred to as exonic splicing enhancers (ESEs) [6]. In contrast, hnRNPs frequently interfere with the assembly of the spliceosome by interacting with exonic or intronic *cis*-elements, also referred as exonic or intronic splicing silencers (ESSs/ISSs) [15]. Even though the impacts of SRSFs and hnRNPs on alternative splicing regulation are context-dependent, these two families frequently constitute an antagonistic mechanism for selecting regulated exons [16]. Under different conditions, the various levels or activities of SRSFs and hnRNPs in nuclei compose the “splicing code” which specifically determines the splicing profile generated from a single pre-mRNA [17]. Therefore, impaired expression of a splicing factor and a mutation within the *cis*-element frequently lead to pathological alterations to AS events. Insertion of DNA transposable elements (TEs), such as *Alu* element, alters ordinary splicing profiles by creating alternative polyadenylation sites, by modulating exonic utilization, and by inducing exonization [18]. For instance, *Alu* and *MER51* elements constitute an antagonistic circuit in manipulating the retention of *ATM* (ataxia-telangiectasia, mutated) intron 28, which activates the alternative splicing-coupled nonsense mediated decay (AS-coupled NMD) pathway [19].

### 1.2. Oncogenic Effects of Dysregulated Splicing Factors

In general, SRSF family members contain one or more RNA recognition motifs (RRMs) and a C-terminal arginine-serine repeat which is referred to as the RS domain [6]. SRSF proteins participate in multiple post-transcriptional regulatory processes, including mRNA export [20], mRNA turnover (Table 1) [21], and constitutive and alternative splicing [22]. Several SRSF proteins were documented to enhance alternatively spliced transcripts which exert pro-oncogenic signatures in distinct cancers [23]. SRSF1 (also referred to as the alternative splicing factor; ASF) is a widely studied protein that is involved in multiple post-transcriptional regulatory processes [24]. SRSF1-regulated splicing events affect a wide range of cellular processes, including cell proliferation, apoptosis, metabolic homeostasis, and related signaling pathways [25]. An increase in SRSF1 expression was frequently identified in different cancers and was linked to its pro-oncogenic potential [25]. SRSF1 was documented to enhance the generation of *cyclin D1^−ex 4^* transcripts, thus encoding the oncogenic isoform cyclin D1b which induces cell proliferation, invasion, and transformation [26]. Upregulated SRSF1 expression mediates a switch in the splicing profiles of *BIN1* and *CASP9* to generate pro-oncogenic isoforms which interfere with the apoptosis of diverse cancer cells [27,28]. An increase in SRSF1 expression was recently found to enhance relative levels of pro-oncogenic isoforms generated from the *Mnk2* and *RON* genes which impair the p38-mitogen-activated protein kinase (MAPK) and mammalian target of rapamycin (mTOR) signaling pathways, consequently enhancing the migratory and invasive activities of different cancer cells [29,30]. Increased expressions of other SRSF family members, including SRSF2 and SRSF3, are pro-oncogenic in specific malignant diseases. SRSF2 (also referred to as SC35) was demonstrated to interfere with the tumor-suppressive signature of the *KLF6* gene by inducing the generation of exon 1a-included transcripts, consequently encoding the DNA-binding domain-deficient isoform [31]. Elevated expression of SRSF3 (also referred to as SRp20) was documented to mediate a shift in the splicing profile of the *MCL-1* gene to the exon 2-containing *MCL-1_l_* isoform, which encodes the antiapoptotic isoform in breast cancer cells [32]. In addition, upregulated expressions of SRPK family members are considered potential oncogenes. Three SRPK family members, SRPK1, SRPK2, and SRPK3, are encoded in mammalian cells. Increases in SRPK1 and SRPK2 were observed in a wide range of distinct cancers, including breast cancer, colorectal cancer, lung cancer, ovarian cancer, hepatocellular carcinoma, pancreatic cancer, leukemia, and gliomas [33,34,35,36]. SRPK family members specifically phosphorylate the serine residue at the serine/arginine dipeptide within the RS domain of SRSF proteins [37], which affects the subcellular distribution and regulatory activity of SRSFs’ post-transcriptional regulation [38]. Increased expressions of SRPK1 and SRPK2 are linked to progressive signatures of carcinogenesis, such as active proliferation and anti-apoptosis, in cancer cells [39,40], whereas altered expressions of SRPK1 and SRPK2 were demonstrated to exhibit both oncogenic and tumor-suppressive effects in in vitro cell and animal models [41]. These results suggest the therapeutic value of the SRPK family for targeting cancer treatment.

The hnRNP family is comprised of 20 members, named A1 to U, in human cells [42]. Six major hnRNP proteins, including A1, A2, B1, B2, C1, and C2, were reported to be associated with nascent pre-mRNA in a bead-on-a-string structure [43]. hnRNPs exert diverse functions involved in post-transcriptional controls, such as capping, splicing, polyadenylation, mRNA transport, and mRNA stability [44]. In addition to RRM, the RGG box containing an Arg–Gly–Gly tripeptide and a triple-repeat KH domain contributes to the RNA-binding capability and specificity of hnRNP family members [45,46]. HnRNPs contain auxiliary proline-, glycine-, or acid-rich domains that are responsible for protein-protein interactions [47]. Deregulated expressions of hnRNPs are frequently noted in distinct types of cancers, which are linked to the proliferation, immortality, angiogenesis, and metastatic activity of cancer cells (Table 1) [48]. Among those members, the impact of hnRNP A1 on post-transcriptional controls, including AS, is widely studied [49]. Impaired hnRNP A1 expression was found in various cancers, including liver, lung, and colon cancers [50,51,52]. Upregulated hnRNP A1 was reported to induce the active metastasis of breast cancer cells by regulating *CD44* splicing [53]. The HOXB-AS3 peptide generated from noncoding RNA suppresses carcinogenic metabolism by interfering with the RNA-binding activity of hnRNP A1 and subsequently reprograms the splicing profile of *pyruvate kinase M*, a key factor involved in the Warburg effect [54]. Elevated expression of hnRNP A2 is widely observed in distinct types of cancer [55,56]. Forced reduction of hnRNP A2 mediated apoptosis and growth arrest of various cancer cells in an in vitro model [57]. HnRNP A2-regulated splicing events modulated activation of RAS-MAPK signaling, which is critical for the epithelial-to-mesenchymal transition (EMT). Overexpression of hnRNP A2 enhanced relative levels of the full A-Raf isoform that is required for activation of the RAS-MAPK pathway in hepatocyte carcinoma cells [58,59]. In addition, overexpressing hnRNP A2 mediated an increase in the constitutively active ΔRON isoform encoded from the alternatively spliced *RON tyrosine kinase receptor* transcript, which is linked to the active RAS-MAPK signaling pathway [60].

### 1.3. Impacts of Dysregulated Splicing Events on Carcinogenesis

Aberrant splicing events are frequently observed in distinct types of cancer cells [61]. Despite the development of high-throughput analyses, such as deep RNA-sequencing (RNA-seq), knowledge regarding the impacts of altered splicing events on carcinogenesis is still limited. In addition to the dysregulated expressions of splicing regulators, mutations within splice sites and regulatory elements constitute another mechanism involved in generating carcinogenesis-related transcripts. Analytical results of RNA-seq across distinct types of cancer from The Cancer Genome Atlas (TCGA) indicated that aberrant intron retention was more frequently identified than other splicing patterns in almost all cancer types [8,62]. Integrated results from whole-genome, whole-exome, and whole-transcriptome assays indicated that somatic mutations were more frequently identified in cancerous tissues than in adjacent normal tissues dissected from the same patient [8,9,62]. Somatic mutations at exon-intron boundaries commonly lead to intron retention that interferes with mRNA splicing [8]. Interestingly, retention of most somatic mutation-mediated introns consequently leads to the generation of premature termination codon-harbored transcripts yielded from tumor-suppressor genes [63]. Retention of *TP53* intron 9 results in the generation of TP53β and TP53γ, which exert discriminative preferences in regulating the transcriptional activities of specific candidates [64,65]. Large proportions of somatic mutations at responsive elements within regulated exons or flanking introns synonymously disturb splicing profiles [8,9]. Carcinogenesis-related exonic or intronic mutations are frequently annotated close to exon-intron boundaries within proto-oncogenes, but not tumor-suppressor genes [8]. Nevertheless, these somatic mutations preferentially interfere with the ordinary influence of *cis*-splicing elements of utilizing regulated exons.

## 2. Therapeutic Strategies Targeting Alternative Splicing Events in Cancer

The specificity or severity of cancer-associated splicing events suggests their therapeutic potential against carcinogenesis. Accordingly, several strategies from conventional small-molecule compounds, gene silencing, and gene editing are proposed for treatment development.

### 2.1. Targeting the Core Spliceosome Machinery

An identified somatic mutation of the core spliceosome was demonstrated and considered a preferential target to cause lethality of cancer cells with splicing abnormalities [66,67]. Three types of small compounds derived from fermentation products of distinct bacteria were demonstrated to exert anticancer properties by mainly targeting the SF3B component of U2 snRNP and subsequently impairing the spliceosome assembly. These natural compounds or derived analogs include spliceostatins derived from *Pseudomonas*, and herboxidienes and pladienolides derived from *Streptomyces*, which show potent cytotoxicities to mediate cell cycle arrest at the G_1_ and G_2_/M phases in cell and animal models (Table 2) [68,69,70]. Recent studies documented that the derived analogs of these small compounds, such as pladienolide E7107, spliceostatin A, and sudemycin D6/K, have improved stability and reduced half-maximal inhibitory concentrations and may be more suitable for therapeutic applications (Table 2) [71,72,73]. A growing body of RNA-seq results indicates that sequence variations close to the 3′ splice site exert critical influences on diverse splicing profiles, which consistently suggests the therapeutic potential of SF3B-targeting compounds against cancer [73]. For instance, recent reports documented that MYC-dependent cancers are preferentially sensitive to a modulator of the spliceosome component, including SF3B [74]. Intriguingly, copy loss of the wild-type *SF3B* gene was demonstrated to facilitate sensitivity to spliceosome-targeting therapy [75]. In addition, several compounds were identified that target other components of the spliceosome. Rearrangement of the spliceosome during RNA splicing is orchestrated by Brr2 of U5 snRNP, an ATP-dependent RNA helicase, to trigger unwinding of the U4/U6 RNA duplex (Table 2) [76]. High-throughput screening was recently conducted to identify potential compounds that preferentially target Brr2 [77]. Although the impacts of these small molecules on the RNA splicing of cancer cells have still not been determined, targeting RNA helicases could be a potential therapy to induce vulnerability of cancer cells.

### 2.2. Targeting Splicing Regulators in Cancer

In vitro preclinical studies identified that increases in splicing regulators are linked to active signature of carcinogenesis, which highlights the therapeutic potential of targeting these factors. In particular, upregulated expression of SRPK1 is frequently considered to be an oncogenic factor in a wide range of cancer types [41]. By treating kinase inhibitors of the SRPK, CLK, and DYRK families, such as TG-003 and SRPIN340 (Table 2), reduced phosphorylation of SR proteins was linked to an AS-coupled NMD mechanism that contributes to reduced *SRSF2*, *FAS,* and *VEGF* transcripts in distinct cancer cells [78,79,80,84]. A series of related compounds, including Cpd-1, Cpd-2, and Cpd-3, that interfere with the activities of the SRPK and CLK families was recently identified by large-scale screening [80]. Cpd compounds are characterized as exerting polished specificity targeting the SRPK and CLK families, even though the molecular mechanism remains uncharacterized (Table 2) [84]. Moreover, an orally bioavailable and metabolically stable compound (TG693) was shown to potentially inhibit excessive SRPK and CLK activities in limited types of cancer cells; a preclinical test in a bigger panel of cancer cells is required to verify the therapeutic potential [85].

### 2.3. Oligonucleotide-Based Therapy to Modulate Splicing in Cancer

Compared to the extensive impacts and side effects that are elicited by small molecules and derived compounds, there is sustained interest in developing an oligonucleotide-based modality to target carcinogenesis-related splicing transcripts with high selectivity and sensitivity. Engineered RNA oligonucleotides that hybridize to splicing sites, *cis*-regulatory elements, and alternatively spliced transcript are the most common approach to reprogram splicing profiles [86]. Among the strategies, a splice site-switching oligo (SSO) is synthesized RNA that specifically hybridizes with the splice site and *cis*-element, thus consequently interfering with the interplay of *trans-*acting factors with the responsive element [87]. The SSO approach was documented to exert pro-apoptotic and chemosensitizing effects on a wide range of cell models, especially glioma cells, by shifting *Bcl-x* pre-mRNA splicing from the *Bcl-x_l_* to the *Bcl-x_s_* transcript [88]. The 2′-*O*-methoxyethyl-phosphorothioate-modified *Bcl-x* SSO was designed as a base-pair of the 5′ alternative splice site of *Bcl-x* intron 2. Targeting SSOs to the *cis*-element preferentially recognized by the mutant SRSF2 alone suggests its therapeutic potential toward SRSF2-mutated malignancies [89,90,91]. Similarly, SSO-mediated interference of the binding of SRSF1 or hnRNPA1 to the responsive element identified using nucleotide resolution crosslinking immunoprecipitation (iCLIP) and the subsequent effect on the reprogramming splicing profile suggest a potential application to specifically target cancer-related splicing events [92,93]. In addition, the application of antisense oligos (ASOs) is another strategy to eliminate cancer-specific transcripts through RNA surveillance. In spite of the use of ASOs having been approved by the US Food and Drug Administration for Duchenne muscular dystrophy (Eteplirsen 1) and spinal muscular atrophy (Nusinersen 1) [94,95], the application of ASOs and SSOs to cancer therapy is still under investigation and evaluation. Recently, an in vitro cell model identified the repressive effect of an ASO (AZD9150) on reducing signal transducer and activator of transcription 3 (STAT3) expression by directly targeting its transcripts, which exerts antitumor impacts on lung cancer and lymphomas (Table 2) [82]. The application of another ASO (AZD4785) which targets the *KRAS* gene was demonstrated to diminish the proliferative activity of KRAS-driven cancer types (Table 2) [83]. ASO-mediated exclusion of *MDM4* exon 6 leads to a decrease in MDM4 abundance through the AS-NMD pathway, which enhances the drug sensitivity and apoptosis of melanoma cells (Table 2) [81]. These results reveal potential opportunities for directly eliminating cancer-specific transcripts instead of redirecting the splicing profile for therapeutics of malignant diseases. Nevertheless, a specific delivery system against malignant cells remains a major challenge in the development of oligonucleotide-based therapies.

## 3. Conclusions

A growing body of evidence demonstrates that dysregulation of AS events can function as biomarkers and therapeutic targets for diverse types of cancers. A wide range of therapeutic strategies are applied to reprogram splicing events linked to cancer pathologies. Progression in realizing the regulatory mechanism(s) involved in cancer-associated splicing events will help to develop appropriate and specific therapeutic treatments for different cancer types. In the era of high-throughput analysis, whole transcriptome sequencing is considered another critical technique to utilize in selecting personalized therapies for individual cancer patients.

## Figures and Tables

**Table 1:** The influences of splicing factors in cancer cells.

Splicing Factor	Cancer Type	Target Gene	Cellular Effect	Reference
SRSF1	Breast, Lung, and Colorectal cancer	*Cyclin D1*	Upregulated cyclin induces cell proliferation, invasion, and transformation	[26]
		*CASP9* *BIN1*	Reduced apoptosis	[27,28]
		*Mnk2* *RON1*	Active migration and invasion of different cancer cells	[29,30]
SRSF3	Breast cancer	*MCL-1*	Reduced apoptosis of cancer cells	[32]
hnRNP A1	Liver, lung, and colon cancer	*CD44* *Pyruvate kinase M*	Active metastasis and proliferation Upregulated Warburg effect	[53,54]
hnRNP A2	Diverse cancer types	*RAS* *RON*	Active metastasis and proliferation	[58,59,60]

**Table 2:** Small compounds and oligonucleotides documented to alter splicing events.

Type	Compound	Target	Mechanism	Effect on Splicing or Phenotype
Small molecule	Pladienolides E7107 (Derived compound of Pladienolides A–G)	SF3B1 [71]	Abolish the conformation rearrangement of SF3B1	Interfere with canonical splicing Cell cycle arrest
	Herboxidienes	SF3B1 [69]		
	Spliceostatins (FR901463, FR901464, FR901465, FR901464, Sudemycin D6/K; [66,67,68,69,70,71])	SF3B1 [68]		
	Brr2	U5 snRNP [76,77]	Interfere with the RNA helicase activity	Stall canonical RNA splicing
Protein inhibitor	TG-003; TG-693	CLK family [78]	Interfere with ATP binding	Reduced phosphorylation of SRSF family members Altered cellular localization of SRSFs
	SRPIN340	SRPK family [79]	ATP binding competitor	
	Cpd-1/2/3	SRPK and CLK family [80]	ATP binding competitor	
Oligonucleotide	ASO-MDM4	MDM4 transcript [81]	mRNA degradation	Reduce expression of MDM4 mRNA
	AZD9150	STAT3 transcript [82]		Reduce expression of STAT3 mRNA
	AZD4785	KRAS transcript [83]		Reduce expression of KRAS mRNA
